# Allergies and Diabetes as Risk Factors for Dengue Hemorrhagic Fever: Results of a Case Control Study

**DOI:** 10.1371/journal.pntd.0000699

**Published:** 2010-06-01

**Authors:** Maria Aparecida A. Figueiredo, Laura C. Rodrigues, Maurício L. Barreto, José Wellington O. Lima, Maria C. N. Costa, Vanessa Morato, Ronald Blanton, Pedro F. C. Vasconcelos, Márcio R. T. Nunes, Maria Glória Teixeira

**Affiliations:** 1 State University of Bahia, Salvador, Brazil; 2 London School of Hygiene & Tropical Medicine, London, United Kingdom; 3 Federal University of Bahia, Salvador, Brazil; 4 State University of Ceará, Fortaleza, Brazil; 5 Case Western Reserve University, Cleveland, Ohio, United States of America; 6 Evandro Chagas Institute, Pará, Brazil; Pediatric Dengue Vaccine Initiative, United States of America

## Abstract

**Background:**

The physiopathology of dengue hemorrhagic fever (DHF), a severe form of Dengue Fever, is poorly understood. We are unable to identify patients likely to progress to DHF for closer monitoring and early intervention during epidemics, so most cases are sent home. This study explored whether patients with selected co-morbidities are at higher risk of developing DHF.

**Methods:**

A matched case-control study was conducted in a dengue sero-positive population in two Brazilian cities. For each case of DHF, 7 sero-positive controls were selected. Cases and controls were interviewed and information collected on demographic and socio-economic status, reported co-morbidities (diabetes, hypertension, allergy) and use of medication. Conditional logistic regression was used to calculate the strength of the association between the co-morbidities and occurrence of DHF.

**Results:**

170 cases of DHF and 1,175 controls were included. Significant associations were found between DHF and white ethnicity (OR = 4.70; 2.17–10.20), high income (OR = 6.84; 4.09–11.43), high education (OR = 4.67; 2.35–9.27), reported diabetes (OR = 2.75; 1.12–6.73) and reported allergy treated with steroids (OR = 2.94; 1.01–8.54). Black individuals who reported being treated for hypertension had 13 times higher risk of DHF then black individuals reporting no hypertension.

**Conclusions:**

This is the first study to find an association between DHF and diabetes, allergy and hypertension. Given the high case fatality rate of DHF (1–5%), we believe that the evidence produced in this study, when confirmed in other studies, suggests that screening criteria might be used to identify adult patients at a greater risk of developing DHF with a recommendation that they remain under observation and monitoring in hospital.

## Author Summary

Dengue is an arboviral disease that affects large areas of countries in tropical and subtropical regions of the world. Around 500,000 cases and 22,000 deaths of dengue hemorrhagic fever (DHF)/Dengue Shock Syndrome (DSS), the most severe presentations of this disease, occur annually. It is unclear why some cases of dengue fever (0.5% to 4%) progress to DHF/DSS. There is weak evidence that some diseases could have a role in this process, such as diabetes, hypertension, and allergies. In epidemics most dengue fever cases are sent home as there are too many to be kept in observation, but if it were possible to identify those with a higher risk of progression to DHF, they could be kept for observation, for early detection of signs, symptoms and alterations in laboratory tests suggestive of DHF, to enable timely and effective clinical management and early intervention. We study this issue and we believe that the evidence produced in this study, when confirmed in other studies, suggests that screening criteria might be used to identify adult patients at a greater risk of developing DHF with a recommendation that they remain under observation and monitoring in a hospital.

## Introduction

Dengue is the most important viral vector-transmitted disease worldwide in terms of the total number of cases, disease morbidity and mortality [1]. This arboviral disease affects large areas of countries in tropical and subtropical regions of the world. As the physiopathology of the severe presentations, dengue hemorrhagic fever (DHF)/Dengue Shock Syndrome (DSS), remains poorly understood, there are no effective means to predict or prevent progression to this severe clinical expression of the infection [2], [3].

According to the World Health Organization (WHO) [4], around 500,000 cases of DHF/DSS, occur annually, with 22,000 deaths. Until the 1980's years, Southeast Asia was the region most affected by dengue; then it spread to Central and South America and now it is present from the 35^th^ parallel north to the 35^th^ parallel south [2]. In Southeast Asia the incidence of DHF/DSS is high and the disease typically affects mainly children [5]. Until 2007, the clinical pattern of dengue was very different in Brazil (currently the country that reports most cases of dengue fever to the WHO): the majority of cases had occurred among adults and the percentage of the severe forms of the disease was low; but since the 2008 epidemic in Rio de Janeiro, the risk of morbidity and mortality due DHF/DSS began to rise in children [6], [7].

It is not clear why some cases of dengue progress to DHF; understanding this process is essential for preventing it. There is evidence that sequential infections by different dengue viral serotypes plays a important role [8]. However, even during widespread epidemics of dengue fever, in populations with high levels of antibodies against dengue virus (indicating a previous infection), the proportion of cases that progress to DHF is small, ranging from <0.5% to 4% of all cases [9], [10] indicating that other factors are involved in disease severity. Several hypotheses have been raised but so far none supported by solid evidence. It has been suggested that some preexisting chronic diseases such as diabetes, hypertension and bronchial asthma increase the risk of progression to severe forms of dengue [11], [12] based on case series with no control group. The objective of the case-control study reported here was to evaluate the contribution of comorbidities to the development of DHF.

## Methods

This is a matched case-control study carried out in two coastal cities in the northeast of Brazil, Salvador and Fortaleza, with populations of roughly two and a half million each. The minimal sample size (159 cases and 636 controls) was determined to be able to detect an increase of 2.3 fold in the risk of DHF in cases of dengue who also had diabetes (prevalent in 7% of the controls, the least common of comorbidities studied), with 95% precision, 80% power and a ratio of at least 4 controls for each case. We eventually studied 170 cases and 1,175 controls.

The study population consisted of subjects with a history of dengue, confirmed serologically. Only cases and controls who tested positive (IgG) for the anti-dengue antibodies were included in the study, since we wanted to investigate reasons for progression to DHF, not for infection with dengue virus. Cases were selected among those notified with dengue hemorrhagic fever during the period 2002 and 2003 in Salvador and 2003–2005 in Fortaleza. In Salvador the research was conducted in 2004, while in Fortaleza, it was conducted in 2005.

Selection of cases: Cases of DHF registered in the national surveillance system (SINAN) in residents of these two cities between 2003 and 2005 were identified as potential cases for the study. Their surveillance records were examined by two physicians and included in the study if they met the criteria for DHF used by Brazilian Health Service [13] which is very similar to the WHO [14]: (i) fever and positive serology for anti dengue virus IgM and/or viral isolation and characterization by cell culture or RT-PCR (ii) at least two signs or symptoms of dengue fever (headache or retroorbital pain, myalgia, arthralgia, prostration, exanthema) and (iii) all of the following signs: a) hemorrhagic manifestations (at least one type); b) hemoconcentration with an increase of at least 10% in basal hematocrit level (in 80% of the selected cases the increase was 20% or greater, in 13% between 12 and 18% and in 7% between 10 and 12%) and/or hematocrit values >38% in the case of a child, >40% in the case of an adult female and >45% in the case of an adult male; c) thrombocytopenia (≤100,000/mm^3^). We did not consider ascites, pleural effusion, as these were very rarely recorded in the SINAN at the time. In Salvador, of the 91 cases of DHF notified to SINAN, 26 did not meet the criteria and were not included in the study and 65 met the criteria and were invited to participate in the study; 55 agreed to participate. No deaths from DHF were recorded during the study period. In Fortaleza, 194 cases of DHF were notified to SINAN, 55 did not meet the criteria and 139 were invited to participate in the study. Of these 5 had died from others causes since the notification, and 19 refused to participate. The remaining 115 were included in the study. Cases which were excluded had severe dengue but did not meet the criteria for DHF or their records did not have sufficient information.

For each case, a pool of 7–8 potential controls were selected from individuals living in the neighborhood of each case, according to a rule (from the first case on either side of the case's residence, until a suitable control was found, limited to the block) matched by sex and age (within five years), and who reported dengue fever in the same year as the case. Blood was drawn from the pool of potential controls by venous puncture for exclusion of those without seropositivity for dengue. The serum was separated by centrifugation and stored at −20°C. The Department of Arbovirology and Hemorrhagic Fevers of the Evandro Chagas Institute performed ELISA for anti-dengue IgG antibodies, with titers were above 1∶40 [15] being considered positive. 64 potential controls (5.2%) did not have anti-dengue IgG antibodies titers above 1∶40 and were excluded from the study. 1,175 controls remained in the study.

Cases and controls were interviewed (during 2005–2006) at home by teams of trained interviewers using a previously tested, standardized questionnaire, collecting the following types of information: demographic and biological (name, address, age, sex), socio economic (years of schooling, family income expressed as a multiple of the legal minimum salary (US$120 a month at the time of the interview) and self-reported skin color. Clinical information collected included signs and symptoms of dengue and reported morbidity with respect to diabetes, hypertension, allergy, asthma, kidney failure, liver failure and sickle-cell anemia at the time of the reported dengue, and use of medication for these illnesses at the moment of the interview. When the individual reported having had one of these conditions, he/she was asked whether the diagnosis had been made by a doctor and the interviewer asked to see the prescription and/or packaging of any medication. To avoid bias, interviewers were blind to the study objectives and all field work was supervised.

Analysis: To reduce the data, cases and controls were grouped according to their biological and social characteristics and the frequency of self-reported chronic diseases. Reported kidney failure, liver failure and sickle-cell anemia were not considered in this analysis because they were either not reported or reported by very few cases or controls.

The crude association (OR and 95%CI) between the predictors of interest, presence of co-morbidities (hypertension, diabetes, allergy and asthma) and outcome of interest, presence of DHF was investigated using cross tabulations based on matched pair analysis, the McNemar's test [16] (since this was a matched study). Adjusted measures for the association were estimated using multivariate logistic regression adjusting for potential confounding variables. As this was a matched case control study using individual matching, conditional logistic regression was used to estimate the association between the predictors of interest (independent variables: hypertension, diabetes, allergy and asthma) and the occurrence of DHF (dependent variable: presence or absence of DHF) within each matched set of case and controls. Since the study was matched for age, sex, neighborhood and city, the conditional logistic regression adjusted for these variables as potential confounders. To establish whether skin color, schooling and income were also potential confounding variables, we explored the association between these variables and DHF. The subsequent regression models exploring the association between comorbidities and DHF included those variables as potential confounders. A separate model was built for each of the co-morbidities of interest and also for each co-morbidity stratified by number and type of drug used. We did not include a model with all co- morbidities because we did not have sufficient power. Since black skin color has been reported to be a protective factor against DHF [17], [18], and is also known to be associated with hypertension [12], a specific analysis was carried out on cases and controls who described themselves as being black. The STATA® software program, version 9, was used to perform data processing and analysis.

Ethical approval was granted by the Research Ethics Committee, Instituto de Saúde Coletiva, Federal University of Bahia, Salvador, Brazil. Cases and controls who agreed to participate in the study gave written informed consent.

## Results

Of the 170 cases of DHF included in the study, 55.9% were women and around 80% occurred in individuals over 15 years of age. With respect to skin color, 43.5% of cases considered themselves white, 50.0% mixed race and 6.5% black. Over 51% of cases had 11 years or more of schooling and 47.1% had an income of at least four minimum salaries. Of the 1,175 controls, 54.6% were female, 80.9% were over 15 years of age; 27.1% considered themselves white, 64.5% mixed race and 8.4% black; 31.5% had 11 years or more of schooling; and 17.5% had an income of at least 4 minimum salaries. Of these demographic variables, statistically significant differences (p<0.05) were found between the two groups only with respect to skin color, years of schooling and family income. Statistically significant differences (p<0.05) were also found between the cases and controls with respect to self-reported diabetes and allergy (Table 1).Skin color, schooling, and income were all independently associated with DHF. The likelihood (crude, matched OR) of a white individual having been affected by DHF was 4.60 times higher than that of a black participant. Family income ≥4 minimum salaries (OR = 7.02) and ≥11 years of schooling (OR = 4.66) were also found to be risk factors for this clinical form of dengue. There was little variation in the values of these measurements of association following adjustment for chronic diseases, differences remaining statistically significant (Table 2).

**Table 1:** Distribution of Socio-demographic characteristic and morbidities conditions by cases (DHF) and controls (DF).

*Exposure*	*Cases (n = 170)*		*Controls (n = 1,175)*		p value
	*N°*	*%*	*N°*	*%*	
Sex					
Male	75	44.1	534	45.4	
Female	95	55.9	641	54.6	0.74
Age					
≤15 years	35	20.6	224	19.1	
≥16 years	135	79.4	951	80.9	0.64
Skin colour					
Black	11	6.5	99	8.4	
Mixed	85	50.0	758	64.5	**0.01**
White	74	43.5	318	27.1	
Schooling (years of study)					
≤3	19	11.1	210	17.9	
4–7	27	15.9	333	28.3	**0.01**
8–10	37	21.8	263	22.3	
≥11	87	51.2	369	31.5	
Income (minimum salary)					
≤1	38	22.4	471	40.1	
2–3	52	30.5	498	42.4	**0.01**
≥4	80	47.1	206	17.5	
Hypertension					
Yes	20	11.8	148	12.6	
No	150	88.2	1,027	87.4	0.75
Diabetes					
Yes	09	5.3	31	2.6	
No	161	94.7	1,144	97.4	**0.05**
Allergy					
Yes	58	34.1	279	23.4	
No	112	65.9	896	76.3	**0.04**
Asthma					
Yes	10	5.9	62	5.3	
No	160	94.1	1,113	94.7	0.74

DHF – Dengue hemorrhagic fever.

DF – Dengue fever.

**Table 2:** Crude and adjusted* Odds Ratio of the association of Dengue Hemorrhagic Fever with skin colour, schooling and income.

*Exposure*	*OR crude*	*95% CI*	*OR adjusted*	95% CI
Skin colour				
Black	1	1	**1**	**1**
Mixed	1.88	0.91–3.89	1.96	0.94–4.10
White	**4.60**	**2.13–9.90**	**4.70**	**2.17–10.20**
Schooling				
≤3 years	1	1	1	1
4–7 years	1.07	0.55–2.08	1.09	0.55–2.15
8–10 years	**2.35**	**1.17–4.74**	**2.45**	**1.20–4.99**
≥11 years	**4.66**	**2.37–9.15**	**4.67**	**2.35–9.27**
Income (minimum salary)				
≤1	1	1	1	1
2–3	**1.70**	**1.06–2.72**	**1.70**	**1.06–2.72**
≥4	**7.02**	**4.21–11.6**	**6.84**	**4.09–11.43**

*Adjusted Odds Ratio were obtained from a multivariate conditional logistic regression with skin color, schooling, and income being adjusted by selected self-reported chronic conditions.

When each one of the self-reported chronic diseases was adjusted for the ethnic and social variables, only diabetes remained associated with DHF (adjusted OR = 2.75; 95% CI: 1.12–6.73) (Table 3).

**Table 3:** Crude and adjusted* Odds Ratio of the association of Dengue Hemorrhagic Fever with chronic co-morbidities.

*Exposure*	*Cases*	*Controls*	*Matched OR crude* **	*95% CI*	*OR Adjust*	*95% CI*
Hypertension						
No	150	1,027	1		1	
Yes	20	148	0.90	0.50–1.62	0.93	0.51–1.70
Diabetes						
No	161	1,144	1		1	
Yes	9	31	**2.46**	**1.03–5.87**	**2.75**	**1.12–6.73**
Allergy						
No	112	896	1		1	
Yes	58	279	**1.59**	**1.11–2.28**	1.29	0.87–1.89
Asthma						
No	160	1,113	1		1	
Yes	10	62	0.93	0.46–1.89	0.87	0.41–1.84

*Adjusted Odds Ratio were obtained from a multivariate conditional logistic regression with hypertension, diabetes, allergy, and asthma being adjusted by selected self-reported skin color, income and education.

**Matched OR and 95 CI calculated using McNemar's test.

When the self-reported diseases were classified according to use of the respective medications, with the category “absence of disease” as reference, the use of medication (steroids and no steroids) by allergic individuals was found to be positively associated with DHF; however, following adjustment for the ethnic and social variables, the association was maintained only for allergy treated with steroids (OR = 2.94; 95%CI: 1.01–8.54). Of the individuals who reported hypertension, an increasing gradient was found in the crude and adjusted OR when the use of medication was considered (no treatment, treatment with only one antihypertensive drug or treatment with more than one hypertensive drug); however, these differences were not statistically significant (Table 4).

**Table 4:** Crude and adjusted* Odds Ratio of the association of Dengue Hemorrhagic Fever with chronic co-morbidities stratified by number or type of drugs used.

Exposure	Cases	Controls	Matched OR crude**	95% CI	OR Adjust	95% CI
No hypertension	150	1,027	1	1	1	1
Hypertension no drugs	04	57	0.48	0.16 1.38	0.50	0.16 1.49
Hypertension with 1drug	08	55	0.95	0.39 2.27	0.97	0.39 2.41
Hypertension with >1 drug	08	36	1.76	0.74 4.19	1.67	0.68 4.09
No diabetes	161	1,144	1	1	1	1
Diabetes no drugs	01	07	1.55	0.18 13.3	1.83	0.18 18.67
Diabetes 1 drug	05	15	2.32	0.74 7.24	2.72	0.86 8.60
Diabetes with insulin or more than 1 drug	09	03	3.53	0.83 14.9	3.36	0.72 15.61
No allergy	112	896	1		1	
Allergy no drugs	44	235	1.46	0.98 2.16	1.16	0.76 1.77
Allergy drugs anti allergic	08	27	1.90	0.80 4.55	1.45	0.58 3.61
Allergy drugs steroids	06	17	**2.79**	**1.05 7.44**	**2.94**	**1.01 8.54**
No Asthma	160	1,113	1		1	
Asthma no drugs	05	42	0.63	0.23 1.67	0.60	0.21 1.66
Asthma drug no steroids	04	12	2.29	0.72 7.26	2.13	0.63 7.21
Asthma drug with steroids	01	08	0.71	0.84 5.96	0.60	0.05 6.28

*Adjusted Odds Ratio were obtained from a multivariate conditional logistic regression with hypertension, diabetes, allergy, and asthma being adjusted by selected self-reported skin color, income and education.

**Matched OR and 95 CI calculated using McNemar's test.

In the subgroup analysis of the cases and controls who described themselves as black, the individuals who had hypertension and used more than one antihypertensive medication were found to have a 13-fold greater likelihood of having DHF (95%CI: 1.42–118.8) compared to black individuals who did not have hypertension. Black individuals with hypertension who did not use medication or who used only one type of antihypertensive medication were around four times more likely to have had DHF when compared to black individuals without hypertension; however, this difference was not statistically significant(data not shown on tables).

## Discussion

The results of this study showed that individuals who reported having allergies (and for which they used steroids), or those who reported diabetes, were two and a half times as likely to have DHF. DHF is known to be more common in repeat dengue infections. Current knowledge about the physiopathology of DHF suggests amplification of the immune response due to the presence of heterotypic antibodies against a serotype of the dengue virus at the time of new infection [8], [11] as an explanation for the higher frequency of DHF in repeat dengue infections. The immune system in allergic individuals may be persistently activated with signs of inflammation in tissues and capillaries [19], [20], and if we consider the use of steroids for allergy as a marker of the severity of the allergy, one can conclude that severe allergy is even more likely to lead to inflammation and liberation of pro inflammatory cytokines in tissues, particularly in the endothelium. An alternative explanation for this finding might be that steroid use itself may increase risk of DHF since they can produce capillary fragility. The inflammation hypothesis is consistent also with the increased risk with diabetes. Type II diabetes, a metabolic disorder of adults that reduces the use of glucose by the organism, changes the anatomical and physiological integrity of the endothelium due to a permanent inflammatory condition caused by activation of T-lymphocytes. This process leads to the release of pro-inflammatory cytokines such as gamma interferon (IFNy) and TNFα [21], [22]. These cytokines are known to have a fundamental role in one of the main phenomena responsible for the clinical manifestations of DHF, the third space fluid shift [23], which is a consequence of endothelial dysfunction and results in hemoconcentration, hypotension and shock. It would appears that triggering endothelial dysfunction may be the common biological mechanism by which allergy and diabetes increase the risk of progression to DHF, by increasing the intrinsic permeability of the endothelial surface of hosts who have been previously infected by another serotype, permitting the occurrence of fluid shift.

Although no statistically significant association was found between hypertension and DHF, it is interesting that when individuals without hypertension were taken as a reference group, a clear trend was found for an increased likelihood of DHF among those who reported having hypertension but did not use any antihypertensive medication followed by hypertensive individuals who used more than one antihypertensive drug. On the other hand, the thirteen-fold higher association between individuals who consider themselves black and use an antihypertensive drug and DHF, when compared with non-hypertensive black individuals, strengthens the hypothesis that preexisting diseases in which physiopathology detrimentally affects endothelial function may help trigger the phenomenon of fluid shift resulting from the increased vascular permeability that characterizes DHF.

The increase in DHF in subjects with white skin color is well described [11], [17] and recent *in vivo* study associated these differences with genetic markers of African ancestry [18]. In the cities where the study was conducted the proportion of individuals of African descent (black and mixed) is over 68%, the majority belonging to a lower socioeconomic stratum than the white population, with lower family income and poorer education [24]. The greater likelihood of DHF in white individuals with higher education levels and higher family income is consistent with both a mixed ancestry subjects being more likely to call themselves white if they are rich and well educated or the presence of some other undetected lifestyle factors associated with an increase the risk of DHF.

The growth in the frequency of allergic and atopic diseases that has been observed in recent decades has emphasized the need for research studies capable of explaining the mechanisms involved in this phenomenon. Changes in lifestyle, initially in countries of Eastern Europe, have occurred in parallel with a growth in the rate of asthma and other allergic diseases [25], [26], [27]. There is evidence that individuals living in cleaner environments, who are consequently less exposed to infections, are at a greater risk of developing allergic diseases (“the hygiene hypothesis”). Although this hypothesis has been controversial, there evidence that some infections early in life may protect individuals against allergic and atopic diseases years later [28], [29]. It is therefore feasible to speculate that white individuals with higher education levels and family income, who in this study were found to have a 7-fold greater risk of having been affected by DHF, would also constitute the segment of the population with the highest risk of suffering from allergies or of having higher levels of allergic sensitization, which may act as triggers of the mechanisms of amplification of the immune response. This hypothesis should be the object of new studies designed to clarify the issue.

This study was retrospective, based on reported co-morbidities, and a self-reported date of history of dengue fever in controls. The diagnosis of DHF was based on the information from review of cases investigated by the surveillance system. These potential limitations create methodological concerns. However, to be included in this analyses, available hospital data was reviewed by experts and found to satisfy the clinical and laboratory criteria described in Methods. To increase the validity of reported co-morbidities and medication, the investigators requested to see medical prescriptions and drug packaging to ensure that the information obtained indirectly was as reliable. The data collection procedures were standardized and identical for the two comparison groups.

A potential bias in this study is that dengue infection rates varied in Salvador and Fortaleza by skin color. However, there is good evidence that skin colour is not associated with dengue infection [30]. As to likelihood that a diagnosis and notification of DHF varies by skin color, this is also very unlikely because Brazil has universal health care free at the point of use. As for notifications of cases to health authorities by provider, is known that private health providers less often notify cases to National Health Service than public sector provider. This would tend to introduce a bias toward cases reported among lower incoming and black patients.

Subgroup analysis in blacks had not been planned. We hypothesized that hypertension was a risk and we knew of course that hypertension is more common in blacks. We did the subgroup analysis because hypertension risk was not found to be a risk factor in the role patient population. We suggest a cautious interpretation of this finding but urge that this be investigated in future studies.

We did not present results separately for the two cities. This is appropriate as matching for cities would have controlled for any confounding; it would not have been appropriate if the associations between co-morbidities and DHF were different in the two cities. We did examine the magnitude of the associations in the two cities and judged them to be similar.

The exclusion of individuals who were seronegative under the sample constraints described assured that all cases and controls, had dengue infection in the past. DENV1 and DENV2 have been circulating intensely in the two cities since the 1990s and the populations of these cities have high levels of antibodies against these serotypes [31], [32]. Most cases of DHF included in this study were caused by DENV3, which was introduced into these cities only in 2002 [10]. Because this is a retrospective study, we do not know the date of or number of dengue infections in control. It is possible that many individuals were infected prior to 2005 or 2006. It is important to emphasize that controls reported having had a dengue like illness during the same period of the matched case and reported never having had DHF.

This is the first case-control study to investigate the association between hospitalization with a diagnosis of DHF and evidence of diabetes and allergy, and hypertension and the results reported here are the initial evidence for this very important association. In dengue epidemics most dengue fever cases are seen in out-patients settings and then sent home, as there are too many for all to be hospitalized for observation, in spite of the potential for progression to DHF. If it were possible to identify those with higher risk of progression to DHF, and to keep them for observation, for to early detection of signs, symptoms and alterations in laboratory tests suggestive of DHF, this would enable timely and effective clinical management. Early intervention is such cases will reduce mortality substantially, since the case fatality rate of DHF in SE Asian patients is high (1–5%). We believe the evidence produced in this study when confirmed suggests that screening criteria might be used to identify adult patients at a greater risk of developing DHF with a recommendation that they remain under observation and monitoring in hospital. We also recommend further clinical studies to define new protocols on the evolution of dengue infections in patients with diabetes, allergies and hypertension (particularly with respect to drugs used) and appropriate medical management. Finally, cross immunologic pathophysiologic studies based on the associations between diabetes, allergy and high socioeconomic status and DHF, are urgently needed to investigate the intricate mechanism controlling severe forms of dengue.

## Supporting Information

Checklist S1 STROBE checklist.
(0.20 MB RTF)
