# Supplementary material for: Allergies and Diabetes as Risk Factors for Dengue Hemorrhagic Fever: Results of a Case Control Study
Source: PLoS Negl Trop Dis. 2010 Jun 1;4(6):e699. doi: 10.1371/journal.pntd.0000699 (PMC2879373; doi:10.1371/journal.pntd.0000699)
Supplement: Checklist S1 — STROBE checklist. (0.20 MB RTF) [file pntd.0000699.s001.rtf]

STROBE Statement—Checklist of items that should be included in reports of case-control studies 
	Item No	Recommendation	
Title and abstract
	     (Yes) 1
	(a) Indicate the study's design with a commonly used term in the title or the abstract	
		(b) Provide in the abstract an informative and balanced summary of what was done and what was found	
Introduction	
Background/rationale	(Yes) 2	Explain the scientific background and rationale for the investigation being reported	
Objectives	(Yes) 3	State specific objectives, including any prespecified hypotheses	
Methods	
Study design	(Yes) 4	Present key elements of study design early in the paper	
Setting	(Yes) 5	Describe the setting, locations, and relevant dates, including periods of recruitment, exposure, follow-up, and data collection	
Participants	(Yes) 6	(a) Give the eligibility criteria, and the sources and methods of case ascertainment and control selection. Give the rationale for the choice of cases and controls	
		(Yes) (b) For matched studies, give matching criteria and the number of controls per case	
Variables	(Yes) 7	Clearly define all outcomes, exposures, predictors, potential confounders, and effect modifiers. Give diagnostic criteria, if applicable	
Data sources/ measurement	8*
(Yes for case and controls)	 For each variable of interest, give sources of data and details of methods of assessment (measurement). Describe comparability of assessment methods if there is more than one group	
Bias	(Yes) 9
	Describe any efforts to address potential sources of bias	
Study size	(Yes)10
	Explain how the study size was arrived at	
Quantitative variables	(Yes)11
	Explain how quantitative variables were handled in the analyses. If applicable, describe which groupings were chosen and why	
Statistical methods	12
(Yes)
(Yes)
(Yes)
(Yes)
(Yes)	(a) Describe all statistical methods, including those used to control for confounding	
		(b) Describe any methods used to examine subgroups and interactions	
		(c) Explain how missing data were addressed	
		(d) If applicable, explain how matching of cases and controls was addressed	
		(e) Describe any sensitivity analyses	
Results	
Participants	13*
(Yes)
(- )
(- )	(a) Report numbers of individuals at each stage of study—eg numbers potentially eligible, examined for eligibility, confirmed eligible, included in the study, completing follow-up, and analysed	
		(b) Give reasons for non-participation at each stage	
		(c) Consider use of a flow diagram	
Descriptive data	14*
(Yes)
(Yes)	(a) Give characteristics of study participants (eg demographic, clinical, social) and information on exposures and potential confounders	
		(b) Indicate number of participants with missing data for each variable of interest	
Outcome data	(Yes)15*
	Report numbers in each exposure category, or summary measures of exposure	
Main results	16
(Yes)
(-)
(Yes)
(-)	(a) Give unadjusted estimates and, if applicable, confounder-adjusted estimates and their precision (eg, 95% confidence interval). Make clear which confounders were adjusted for and why they were included	
		(b) Report category boundaries when continuous variables were categorized	
		(c) If relevant, consider translating estimates of relative risk into absolute risk for a meaningful time period	

Other analyses	(Yes)17
	Report other analyses done—eg analyses of subgroups and interactions, and sensitivity analyses
	
Discussion	
Key results	(Yes)18
	Summarise key results with reference to study objectives	
Limitations	(Yes)19
	Discuss limitations of the study, taking into account sources of potential bias or imprecision. Discuss both direction and magnitude of any potential bias	
Interpretation	(Yes)20
	Give a cautious overall interpretation of results considering objectives, limitations, multiplicity of analyses, results from similar studies, and other relevant evidence	
Generalisability	(Yes)21
	Discuss the generalisability (external validity) of the study results	
Other information	
Funding	(Yes)22
	Give the source of funding and the role of the funders for the present study and, if applicable, for the original study on which the present article is based	

*Give information separately for cases and controls.

Note: An Explanation and Elaboration article discusses each checklist item and gives methodological background and published examples of transparent reporting. The STROBE checklist is best used in conjunction with this article (freely available on the Web sites of PLoS Medicine at http://www.plosmedicine.org/, Annals of Internal Medicine at http://www.annals.org/, and Epidemiology at http://www.epidem.com/). Information on the STROBE Initiative is available at http://www.strobe-statement.org.
